# A Novel Brain Stimulation Technology Provides Compatibility with MRI

**DOI:** 10.1038/srep09805

**Published:** 2015-04-29

**Authors:** Peter Serano, Leonardo M. Angelone, Husam Katnani, Emad Eskandar, Giorgio Bonmassar

**Affiliations:** 1 Athinoula A. Martinos Center for Biomedical Imaging, Massachusetts General Hospital, Charlestown, MA, U.S.A; 2 Department of Electrical and Computer Engineering, University of Maryland, College Park, MD, U.S.A; 3 Division of Biomedical Physics, Office of Science and Engineering Laboratories, Center for Devices and Radiological Health, U.S. Food and Drug Administration, Silver Spring, MD, U.S.A; 4 Department of Neurosurgery, Massachusetts General Hospital, Harvard Medical School, Boston, MA

## Abstract

Clinical electrical stimulation systems — such as pacemakers and deep brain stimulators (DBS) — are an increasingly common therapeutic option to treat a large range of medical conditions. Despite their remarkable success, one of the significant limitations of these medical devices is the limited compatibility with magnetic resonance imaging (MRI), a standard diagnostic tool in medicine. During an MRI exam, the leads used with these devices, implanted in the body of the patient, act as an electric antenna potentially causing a large amount of energy to be absorbed in the tissue, which can lead to serious heat-related injury. This study presents a novel lead design that reduces the antenna effect and allows for decreased tissue heating during MRI. The optimal parameters of the wire design were determined by a combination of computational modeling and experimental measurements. The results of these simulations were used to build a prototype, which was tested in a gel phantom during an MRI scan. Measurement results showed a three-fold decrease in heating when compared to a commercially available DBS lead. Accordingly, the proposed design may allow a significantly increased number of patients with medical implants to have safe access to the diagnostic benefits of MRI.

Magnetic resonance imaging (MRI) for patients with neurostimulators: advantages and current limitations. Implanted medical devices such as cardioverter-defibrillators, pacemakers, spinal cord stimulators, and deep brain stimulation (DBS) have become well-accepted therapeutic options to treat a wide range of medical conditions and contribute to improved quality of life^1^. Many patients with implanted devices can benefit from MRI, which is the diagnostic tool of choice for monitoring structural changes in the body, as well as diagnosing many common illnesses including cancer, cardiovascular disease, and trauma. Additionally, functional MRI is becoming more prevalent in assessing brain function and cognitive disorders^2^,^3^. However, approximately 300,000 patients with implanted or partially implanted medical devices are denied MRI each year because of safety concerns^4^. A major concern when performing MRI examinations in patients with electrically conductive implants is the increase in induced currents (“antenna effect”) along conductive leads in the body that are exposed to the radiofrequency (RF) waves of the MRI. The increase in current flow into the tissue at the point of contact with the lead (i.e. the electrodes) causes a large amount of RF energy to be absorbed in the tissue, which in turn causes surges in temperatures that can lead to serious injury^5^,^6^,^7^,^8^,^9^,^10^,^11^,^12^. Temperature increases of up to 25°C were measured near a DBS 3389 lead (Medtronic, Inc., Minneapolis, MN) in an in-vitro gel phantom at 1.5 T MRI^13^. Additionally, increases of up to 30°C were measured with the Medtronic 3389 lead in a swine head at 9.4 T^14^. More importantly, two cases of serious, permanent neurological injury, after MRI exposure at 1.0 T in patients with DBS implants, have been reported^15^,^16^. In both cases the manufacturer guidelines were not followed and in one case the patient developed paralysis following MRI examination^16^. The lack of access to MRI is expensive to society because patients are denied the benefits of screening and accurate diagnosis. A class of implantable devices — defined as “MR Conditional”^17^ — have been shown to pose no known hazards in the MRI environment when operated with specified conditions. For example, the Activa® DBS system (Medtronic, Inc., Minneapolis, MN) is approved for use in MRI^18^ with several conditions^19^, including limited static and gradient magnetic fields, use of low power sequences, and specific RF coils. These conditions, however, are restrictive. The limit for power absorbed by the patient's head is over 30-fold less than typical values allowed, which restricts the number, the type, and quality of MRI scans that can be performed in a given session. The most commonly used transmit body coils are not allowed, excluding the possibility of using MRI to diagnose morbidities in the human torso (e.g., breast cancer, back pain). Additionally, the conditions exclude the use of 3.0 T MRI systems, which are commonly used in clinical^20^ and research applications^21^,^22^.

## A novel technology to allow the safe use of MRI in patients with DBS implants

The safety evaluation of the RF-induced heating injury risks in patients with implanted medical devices undergoing MRI is based on several testing strategies and tools, including pre-clinical (experimental, computational, and animal testing) as well as clinical testing. Experimental testing includes measuring temperature changes near the device while it is implanted in a gel that simulates the electrical and thermal characteristics of the human body^23^. Additionally, computational modeling has been increasingly used to complement experimental testing, as it allows for extensive, cost-effective and systematic analysis of several variables that can influence the amount of current flow into an implant and the amount of energy absorbed by surrounding tissue.

**Figure 1:**
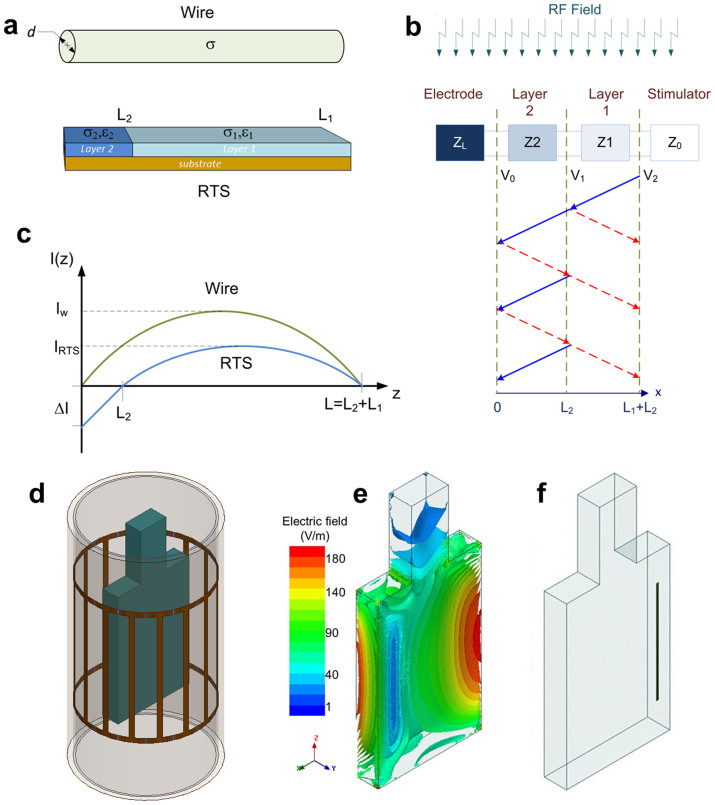
RTS design and simulation setup. (a) Schematic of the PtIr wire (diameter d = 100 μm, electrical conductivity σ = 4.0·10^6^ S/m) and the two-layer RTS design (electrical conductivity σ_1_ and σ_2_, permittivity ε_1_ and ε_2_, length L_1_ and L_2_) used for the study. (b) Equivalent circuit used to model the RTS implant with four sections: stimulator, two layer transmission line, and electrode/tissue interface. The incident RF field induces currents along the implants, which are reflected depending on neighboring sections mismatched impedance (Z_0,_ Z_1_, Z_2_, and Z_L_). The resulting voltage amplitude at each interface (V_0_, V_1_, and V_2_) was generated by the induced current. (c) RF-induced currents along the two leads. The current in the metallic conductor forms a standing wave with high peaks in amplitude (I_w_); conversely, the effect of RTS design is two-fold: a) reduces the average induced currents (I_RTS_) along the implant by worsening the antenna performance, and b) reduces the induced current at the electrode (ΔI) by introducing scattering within the implant. (d) CAD Model used in the numerical simulations, including a 16-leg high-pass birdcage body coil with RF shield, coil former and ASTM phantom. (e) 3 D plot of electric field magnitude at the Larmor frequency (f_0_ = 128 MHz) in the ASTM phantom model used in the simulations. Results were normalized to a power level yielding a whole-body SAR = 2 W/kg (i.e., Normal Operating Mode^41^). (f) Placement of lead inside the phantom. The location was chosen because of the high magnitude of incident electric field.

Several proposals have been made to modify the design of the implant to solve the issue of RF-induced heating without interfering with device performance, such as introducing RF chokes^24^, modifying the materials of the lead (e.g., carbon-loaded leads)^25^,^26^,^27^,^28^,^29^,^30^, or coiling the wire^31^. A new type of lead based on “resistive tapered stripline” (RTS) technology^32^ is herein proposed. The RTS design can be best understood by recalling oceanic science, where an area of study is the prevention of destructive standing waves (clapotis)^33^. Special constructions reinforced with wide, rubble-mound beams break up wave energy over some distance, preventing the formation of clapotis. Similarly, tapered dielectric structures can break up or scatter RF energy due to their unique frequency response characteristics. This characteristic has been studied for many applications including microwave, millimeter-wave and optical-wave engineering^34^,^35^,^36^, as well as stealth aircraft technology^37^. This study presents a two-section stripline-based design (Fig. 1a) with an abrupt transition of electrical conductivity along its length. Contrary to a common metallic wire, this design can break up the induced RF current along the lead (Fig. 1b) caused by the MRI RF coil. Consequently, RF-induced current along the RTS lead is more heterogeneously distributed and significantly reduced at the electrode (Fig. 1c). Please refer to the Supplementary Information for the theoretical background on RTS design.

**Figure 2:**
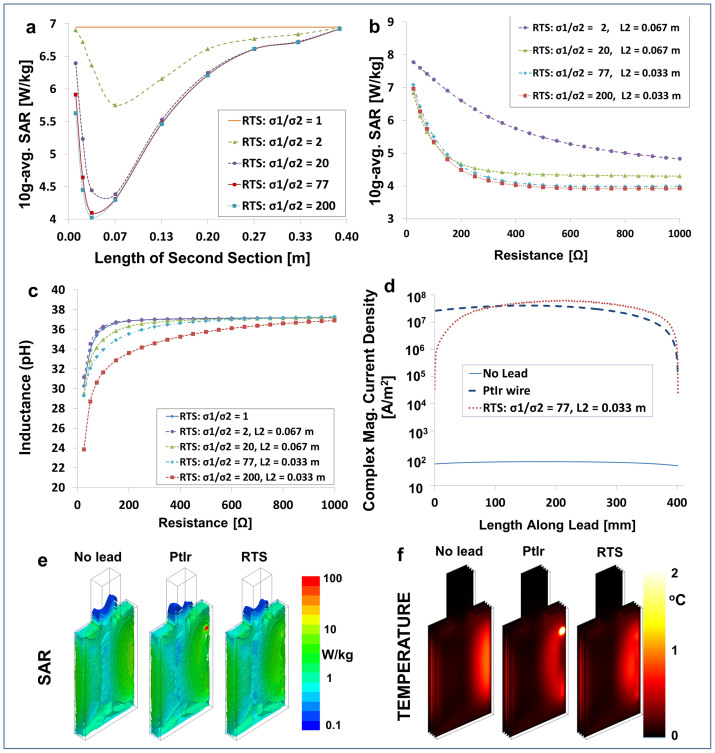
Optimal RTS design in phantom. (a) 10 g-avg. SAR inside the phantom at a distance of 0.1 mm from the electrode obtained by varying the length (L_2_) of the second section (see Methods). Plots include different conductivity ratios for the two layers. In all cases the total resistance of the lead was R = 400 Ω. (b) 10 g-avg. SAR at the same point obtained by varying the total resistance of the lead. Plots include four combinations of conductivity ratios of the two layers and length L_2_ of the second section. (c) Maximum inductance of the RTS varying the total resistance of the lead. Plots include five combinations of conductivity ratios of the two layers and length L_2_ of the second section. (d) Amplitude of induced current inside the lead with the PtIr wire, with the RTS lead selected for prototype manufacturing (right) and in the corresponding volume of the ASTM phantom without lead. The RTS lead allowed for a 37-fold decrease in induced current at the electrode (Length along Lead = 0 mm). In all cases the total length of the leads was 40 cm. (e) Numerical simulation results at 128 MHz calculated with finite element method using the geometry shown in Fig. 1d and 1f and with either a single-electrode PtIr wire or an RTS lead. 10 g-avg. SAR in the ASTM phantom without lead (left), with the PtIr wire (middle), and with the RTS design that was selected for prototype manufacturing (right). Values were normalized to whole-body SAR of 2 W/kg. (f) Temperature maps for 15 minutes of continuous SAR exposure for the same three cases described in (e). Simulations showed that the RTS lead is transparent to the incident RF field and generated similar temperature increase (up to 0.9°C) compared with the ASTM phantom without lead. By contrast, the PtIr wire generated a temperature increase up to 12°C near the electrode (please note that the color bar threshold was set to 2°C to improve visualization.)

**Figure 3:**
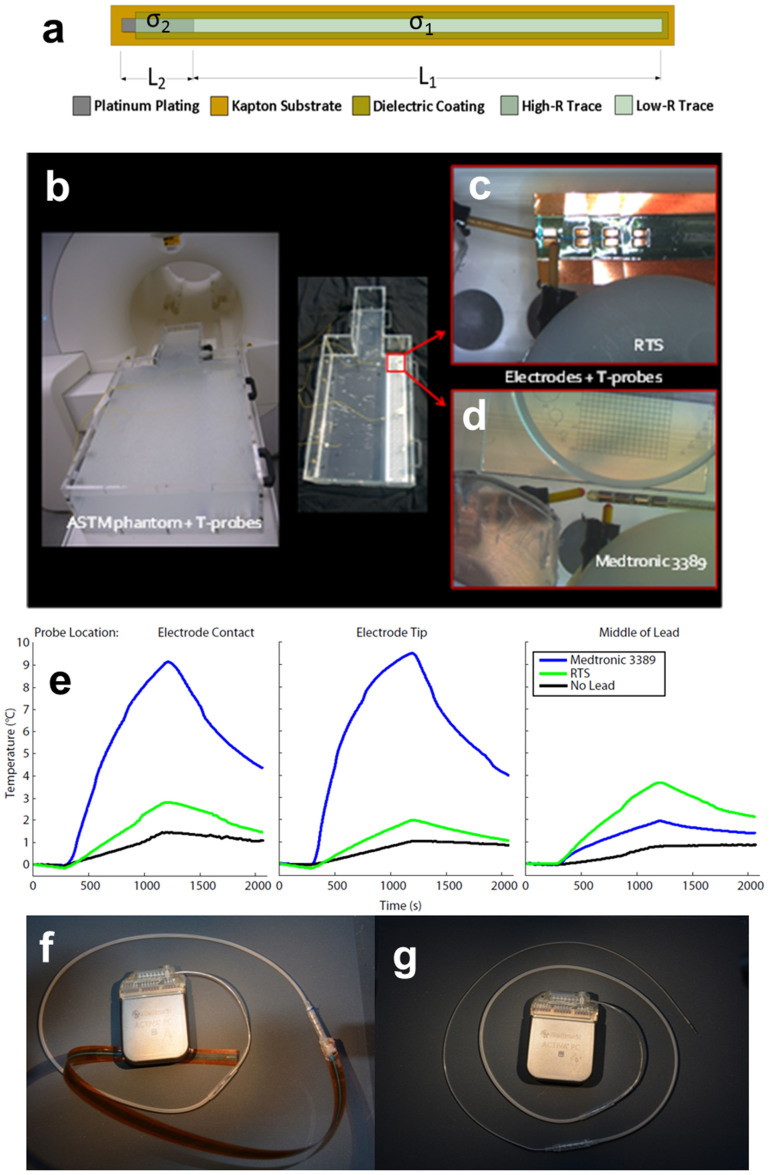
Temperature measurements. (a) Schematic of the two-layer RTS design (electrical conductivity σ_1_ and σ_2_, permittivity ε_1_ and ε_2_, length L_1_ and L_2_ used for the study. (b) ASTM phantom in the 3 T system used for the temperature measurements. The lead was placed laterally on the right side of the phantom, on a white plastic support. (c) Detail showing the temperature sensors placed near the manufactured RTS prototype. The four PtIr electrodes are visible, with the probe located on top of one of them. (d) Commercial lead used for the comparison and placement of the temperature probes near the electrodes. The probes were placed perpendicular to the lead to minimize error accuracy^53^. (e) Results of temperature measurements at three different positions within the phantom without lead, with a Medtronic 3389 lead, and with the RTS lead. (f) Configuration of battery testing with RTS lead. (g) Configuration of testing with the Medtronic 3389 lead. Each of the two leads was connected to a commercial DBS IPG system via an extension. The full system (i.e., IPG, extension, and leads) was immersed in physiologic solution for both the RTS and the commercial lead. Battery consumption was tested over a four-week period for both the Medtronic 3389 and the RTS leads. Both leads showed a 0.005 V initial drop in battery voltage, followed by a constant level over the time evaluated.

**Figure 4:**
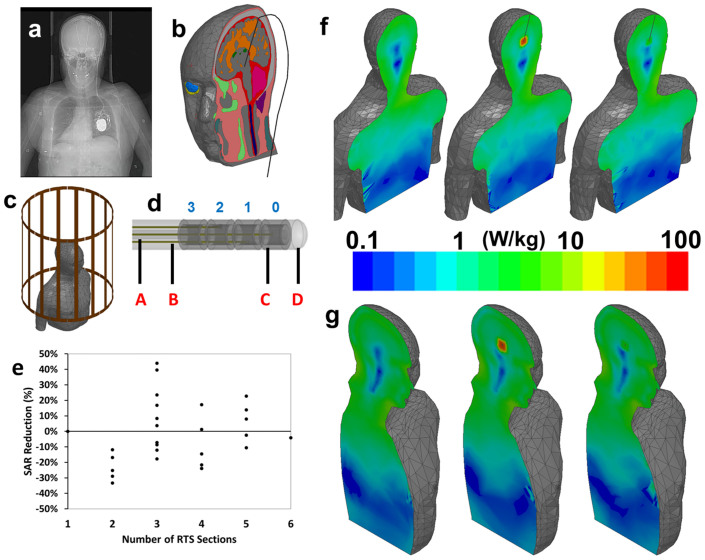
RTS design in human body model. (a) X-Ray image of a patient with implanted bilateral DBS system. A head holder, the implantable pulse generator (IPG) in the thorax, and the two leads are visible. (b) Anatomical model of a human body with implanted DBS lead used for numerical simulations. (c) Model of the human body inside the RF body coil. (d) Model of the lead, including the lumen (A), the four RTS wires (B), the four electrodes numbered as in the Medtronic 3389 (i.e., 0 – 3) (C), and the insulation (D). (e) Graph showing the results of analysis of 10 g-avg. SAR reduction with respect to different RTS configurations (i.e., from two to six sections.) (f) Numerical simulation results showing a coronal view of power absorption in the human body model without implant (left), with PtIr lead (middle), and with RTS lead (right). The increase of power near the electrode for the PtIr lead is clearly visible. By contrast, the RTS wire is “RF-transparent” to the RF field (i.e., is the map is similar to the case without the lead). (g) Sagittal view of the same results.

**Figure 5:**
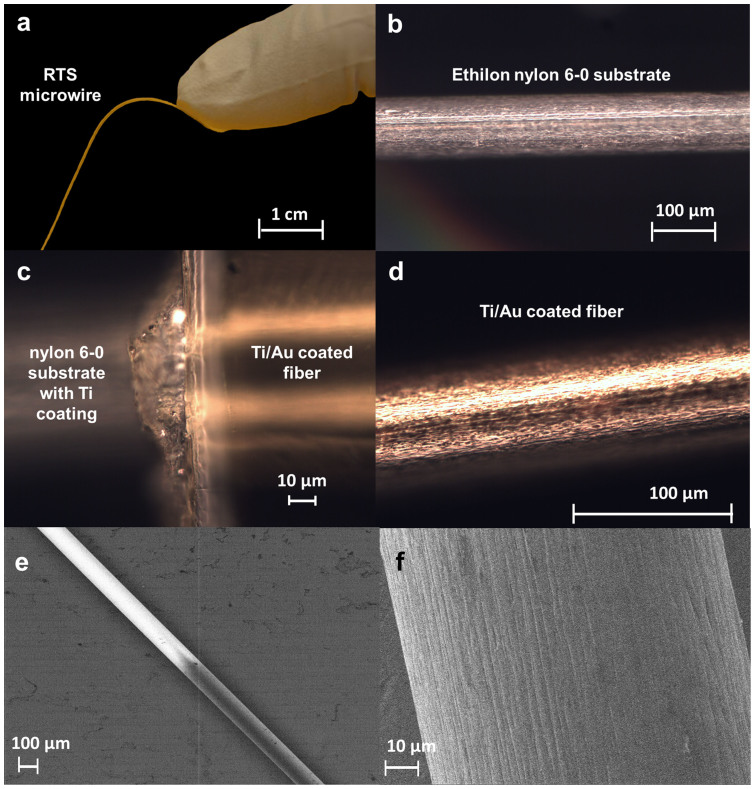
PVD-based manufacturing of wire-design RTS prototype. (a) Two-layer RTS microwire. The RTS (80 μm diameter) was built with an Ethilon nylon 6–0 suture used as a substrate and coated with a Ti/Au layer. (b) Optical microscope (OM) image of the Ethilon nylon 6–0 suture used as a substrate to build the RTS microwire. The steep transition between the two RTS layers — necessary for the maximizing the mismatched impedance and the scattering within the RTS fiber (see Fig. 1b) — is clearly visible. (c) OM image of the interface between the two RTS layers. (d) OM image of microwire fully coated with 100/150 nm of Ti/Au. (e) Scanning Electron Microscope (SEM) image of the RTS microwire. (f) SEM view, with increased magnification, of the Au coating of the RTS fiber showing the characteristic fibrous surface of the Ethilon nylon substrate.

Overall, two different RTS designs were used for the study: (a) an initial design constructed with conductive ink deposited on a polymer substrate (“flat-design”), and (b) a second wire-based design (“wire-design”). The flat-design was used for the simulations in phantom (Figs. 1 and 2), the manufacturing of the first prototype, and the bench testing experiments (Fig. 3). The wire-design was used for simulations with human body models (Fig. 4) and manufacturing of a second prototype (Fig. 5). Both simulations and measurements confirmed that the RTS design “cloaks” the incident RF-field^38^, so that the lead is “RF-transparent” (i.e., the presence of the lead does not significantly affect the RF fields present in a phantom).

## Methods

### Theoretical background on RTS design

The RTS implant exposed to an RF field can be represented with a hybrid model composed of an antenna attached to a transmission line, which consists of resistive traces with sharp changes in conductivity to maximize reflections, followed by a load such as an electrode connected to the tissue (Fig. 1a). As described in ref. 32, the equivalent antenna (i.e., the entire RTS lead) receives the electromagnetic (EM) field and injects it into the first port (i.e., layer) with impedance Z_1_ of such a network (Fig. 1b). A portion of the power transmitted to the first port of the RTS is reflected back as a result of an impedance mismatch between the first port and the antenna, while a remaining portion is supplied to the second layer of the RTS. The impedance of this second port is intentionally mismatched to reflect the greatest amount of power back to the implantable pulse generator (IPG) and away from the electrode that is in contact with the tissue. The fractional power reflected away and delivered to the tissue can be computed from the reflection 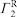 and transmission 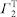 coefficients:
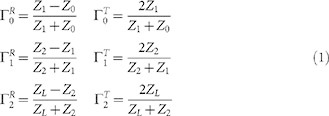
One must consider the superposition of two steady state sine waves in the RTS traveling in opposite directions (Fig. 1b): one forward towards the tissue/electrode (blue) and one backward (red) reflected by the mismatched boundary towards the IPG. The first and second layer of the RTS act both as an antenna and transmission line for the signal that is reflected back away from the tissue/electrode.

The following equation is the typical shape of the ideal current distribution in an RTS wire as sketched in Fig. 1c:
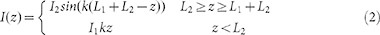
where L_1_ and L_2_ are the lengths of the first and second layer. A more precise current distribution estimated using EM numerical simulations is shown in Fig. 2d. The RTS design reduces the overall inductance of the lead (Fig. 2c). Additionally, the current density has a minimum value along the lead in proximity to the electrode (Fig. 2d), thereby reducing the risk for energy absorption in the surrounding tissue. Please see the Supplementary Information for details on the theoretical background on RTS design.

### Computational modeling and simulations

A computational model was used to evaluate several possible electrical and geometrical configurations of the RTS lead to minimize the absorption of energy and the temperature increase at the electrode. The model included a clinical 3 T MRI RF transmit coil, which operates at 128 MHz^39^, loaded with a gel-filled phantom and an implanted lead (Fig. 1d and 1f). The design contained discrete sections of variable conductivity and length, connected in series, with a fixed length (i.e., to yield a total length of 40 cm to match common lead lengths for implantable devices^13^) and a fixed resistance at low-frequency (i.e., 400 Ω, i.e., less than the typical impedance in patients^40^) (Fig. 2a). Simulations were performed to determine the values of electrical conductivity (i.e., σ_1_ and σ_2_) and length (i.e., L_1_ and L_2_) for a two-section RTS design (Fig. 3a and Fig. 4e) in order to build a prototype for experimental testing. The parameter used in the simulations to evaluate the power absorbed inside the phantom was the specific absorption rate (SAR) averaged over 10 g of tissue (10 g-avg. SAR). SAR (W/kg) is a measure of the energy rate absorbed by the human body when exposed to an RF field and it is the dosimetric parameter used in RF safety guidelines^41^. SAR is averaged either over the entire body, or over 1 g or 10 g of tissue. Temperature simulations on the final optimized lead design were also performed. Please see the Supplementary Information for details on all models and simulations, as well as for additional results.

### Uncertainty analysis

A simulation study to assess the uncertainty of design and simulation parameters was performed (Table 1) following the approach used in Neufeld et al.^42^. Please see the Supplementary Information for details on the methodology used for the analysis.

### Manufacturing of flat-design RTS prototype

Based on the optimal parameters of the RTS design's conductivity and length derived from simulations (see Supplementary Table S1), a flat-design lead prototype was built using polymer thick-film (PTF) technology to experimentally test the proposed concept (see Fig. 3a). The lead was built by printing thin (10 μm) layers of two different commercially available conductive inks on a polymer substrate for the length of each of the two resistive layers. The dimensions of the RTS lead were chosen to obtain the same volume of the wire in the Medtronic 3389 lead (see Supplementary Table S1). Please see the Supplementary Information for additional details on the manufacturing of the two-section flat-design RTS lead prototype.

### Temperature measurements in MRI

The RTS prototype was built by stacking four of the flat-design leads connected to four electrodes (Fig. 3c) and by insulating the proximal end (opposite to the electrodes). The RTS prototype was then implanted in a standard ASTM phantom filled with a polyacrylic acid (PAA) mixed in an aqueous solution^23^ and tested in a 3 T MRI system (Skyra, Siemens, Erlangen, Germany) (Fig. 3b). Three fiber optic temperature probes (Neoptix Inc., Quebec, Canada) were used to record the temperature in the phantom and along the lead under several conditions: no lead, RTS lead, and Medtronic 3389 lead (Fig. 3d). RF energy was delivered to the phantom at First Level Controlled Operating Mode for 15 minutes. Please see the Supplementary Information for additional details on the temperature measurements.

### Implantable pulse generator (IPG) battery testing

Battery testing was also performed with both leads (flat-design RTS and Medtronic 3389) (Fig. 3f and 3g) connected to an IPG (i.e., Medtronic Activa® PC) through an extension. The IPG, extension, and lead were then placed in a quart of deionized water mixed with saline solution to simulate in-body tissue impedance. The IPG was turned on for a total of four weeks. Please see the Supplementary Information for additional details on the IPG battery testing.

### Manufacturing of wire-design RTS prototype

A second RTS wire-design prototype was manufactured using thin-film physical vapor deposition (PVD) of titanium and gold over a rotating Ethilon® 6–0 suture substrate, which was selected for its biocompatibility. Variation in the impedance of each segment is achieved by control of the thickness of the gold layer of each segment. Please see the Supplementary Information for additional details regarding the manufacturing of the wire-design RTS prototype.

## Results

### Electromagnetic simulations

The length of each individual section of the RTS lead affected the 10 g-avg. SAR near the electrode non-linearly (Fig. 2a). Numerical simulations were repeated to observe the correlation between RTS design and 10 g-avg. SAR by fixing the length of each section and varying the ratio in conductivity (σ_1_/σ_2_) between the two sections of the lead, while maintaining a total length of 40 cm. Figure 2b shows the 10 g-avg. SAR in the phantom near the electrode with a resistance varying from 0 Ω to 1 kΩ for several RTS designs. The RTS lead reduced the 10 g-avg. SAR across the entire range of resistances. The simulations showed an increase in conductivity ratio between the two sections that corresponded to a decrease in 10 g-avg. SAR at the electrode. For example, the optimal RTS (i.e., σ_1_/σ_2_ = 200) plateaued at 400 Ω with a value of 4.02 W/kg, whereas the design with σ_1_/σ_2_ = 2 showed a 10 g-avg. SAR of 5.75 W/kg at 400 Ω. For comparison, the peak 10 g-avg. SAR with a 40 cm PtIr wire was 222 W/kg. Please see Supplementary Fig. S1 for additional maps of electric and magnetic field magnitude with RTS and PtIr wire.

The SAR reduction was due to a lower inductance of the RTS design (Fig. 2c) (see also “theoretical background on RTS design” in Methods and Supplementary Information) that corresponded to a shorter equivalent antenna length and lower induced currents. Simulations with a single-section platinum-iridium (PtIr) wire of the same length (40 cm) were also performed for reference. As confirmed by the simulations, the RTS design was characterized by a reduced current at the electrode of over two orders of magnitude compared with the PtIr wire (Fig. 2d). The high electrical conductivity of the ink used for manufacturing allowed a prototype to be built with the following characteristics: σ_1_ = 1.968·10^6^ S/m, σ_2_ = 25.61·10^3^ S/m (i.e., σ_1/_σ_2_ = 76.86), L_1_ = 0.367 m, and L_2_ = 0.033 m. The total resistance for the RTS design was chosen to be R = 400 Ω, five times less than the maximum electrode/tissue impedance of 2 kΩ allowed by even older IPG models^40^. As shown in Figs. 2a and 2b, the 10 g-avg. SAR of this configuration was expected to be very similar to the best performance of the RTS lead with ratio σ_1/_σ_2_ = 200 (i.e., 4.1 W/kg vs. 4.02 W/kg, respectively) (for discussion of the case σ_1/_σ_2_ = 1 as well as other additional cases, please see Supplementary Fig. S2).

### Temperature simulations

Figure 2 shows the 10 g-avg. SAR (Fig. 2e) and temperature maps (Fig. 2f) recorded in the phantom model under three conditions: without implant, with the RTS design selected for prototype manufacturing, and with the PtIr wire. The SAR and temperature maps, which are plotted throughout the plane containing the lead, show similar results between the phantom with the RTS lead vs. the phantom without implant. The peak 10 g-avg. SAR was less than 7 W/kg, and temperature changes were below 1°C in both cases for a 15-minute exposure at a whole-body SAR of 2 W/kg. By contrast, the simulations with the PtIr wire model predicted a peak 10 g-avg. SAR of 230 W/kg and temperature change of 64°C for the same exposure. As a reference, the value of 2 W/kg is the limit in Normal Operating Mode for SAR averaged over the entire body, as established by the current guidelines of the International Electrotechnical Commission (IEC)^41^.

### Temperature measurements

The temperature increase near the electrode of the Medtronic 3389 lead was about 9°C higher than the baseline level of the phantom without a lead and 2°C near the middle of the lead. Conversely, the temperature increase of the RTS lead was 3°C around the electrode and less than 4°C near the middle of the lead (Fig. 3e). These temperature values were consistent with the energy distributions predicted by the simulations (see Supplementary Fig. S3) suggesting a decrease of current at the electrode and an increase of current along the lead. For comparison, the baseline temperature increase of the phantom without an implant was 1.5°C at the location corresponding to the electrode and 1°C at the location corresponding to the middle of the lead (Fig. 3e). Given the linear relationship between SAR and temperature, the corresponding maximum temperature increases at 2 W/kg would be less than 4.5°C with the Medtronic 3389 lead and less than 2°C with the RTS. For reference, the level of temperature increase suggested by an international safety standard for patients with implantable neurostimulators is 2°C^43^, which the RTS lead met in the experimental setup used in this study.

### Battery measurements

The longevity of the Activa PC Neurostimulator battery can last for months to years depending on the following factors: programmed stimulation parameters, the total system impedance and the hours per day the battery is in use. The Medtronic Battery Longevity Manual^44^ provides a formula that estimates the approximate period of time that an Activa PC battery can last. The formula utilizes the aforementioned factors to calculate an estimated energy use of the battery in a 24 hour period, which can then be used with a look-up chart (see Fig. 2 in ref. 44) to predict battery longevity in years. For the battery testing conducted in this investigation, the programmed stimulation parameters, system impedance and hours per day of stimulation were fixed at the same values for both the RTS prototype and the Medtronic 3389 lead. Accordingly, longevity estimates for both leads will be the same. To assess whether actual battery consumption would correspond with such a prediction, a preliminary comparative test was performed by connecting the RTS prototype (Fig. 3f) and the Medtronic 3389 lead (Fig. 3g) to the Activa PC Neurostimulator (Medtronic, Inc., Minneapolis, MN). Over a 30 day testing period, both leads showed only a 0.005 V drop in battery voltage, a result that correlates with Medtronic estimation methods. This indicates that the RTS design affects only the behavior of the lead with respect to RF (i.e., the reactance) and not with respect to the operational frequencies of the stimulator (see also theoretical background on RTS design in Supplementary Information).

## Discussion

This study presents a novel metamaterial^38^ lead that reduces the antenna effect and allows for decreased tissue heating during MRI^45^. The optimal parameters of the design were determined by computational modeling and simulations, validated against in-vitro temperature measurements in a gel-filled phantom (Supplementary Fig. S3). The numerical simulations confirmed that a PtIr wire acts as an antenna during the RF transmit period of the MRI scan, picking up the induced electric field and transferring a high amount of RF energy into the volume surrounding the exposed electrode tip. In both simulations and in-vitro testing the proposed RTS design successfully reduced the amount of energy absorbed and the related temperature increases inside the gel-phantom in proximity to the electrode. Numerical simulations and experimental testing confirmed that the RTS design allows for “RF-cloaking”^38^ while maintaining proper low-frequency conductivity that does not affect battery performance.

The experimental bench testing confirmed also the practical feasibility of the RTS design. The primary feature of the RTS is the abrupt change of conductivity between the two sections. While this discontinuity can be easily modeled computationally, issues can arise in a prototype, because the RTS needs to be built using different inks with different electrical properties. In practice, the two ink traces of the two different layers cannot be perfectly contiguous along the RTS; an overlap is always present which reduces the transition between the layers and, therefore, the ideal step discontinuity in electrical conductivity. The experimental testing confirmed that the prototype contained an adequate discontinuity between the two layers with a physical overlap between the two layers that was only about 50 µm along the RTS (see Fig. 5e). Additionally, the proposed RTS design does not require any external physical device such as an RF choke. RF chokes are difficult to attach to an implant wire because the dimensions of a choke are larger than the typical dimension of the wire. Chokes also disrupt the mechanical characteristics of an implant, which should be flexible^46^. Although there are extremely miniaturized RF chokes, these devices can be more prone to burning because of the microscopic physical dimensions of their components.

In order to enhance the signal-to-noise ratio of the measurements, the testing was performed with high levels of RF power, namely one corresponding to a whole-body SAR of 4 W/kg as estimated by the MRI system. Most sequences used in MRI systems are characterized by a whole-body SAR of less than 2 W/kg. SAR estimation varies for each MRI manufacturer and across systems and depends on several variables, including coil specifications, landmarks, and patient registration information, e.g., weight, height, age. Baker and colleagues^47^ compared the RF-induced heating per unit of SAR due to the presence of a DBS lead between two 1.5 T MRI systems and observed values ranging from 3.5 to 5.5 times higher on one MRI system as compared to the other. As such, the absolute temperature values found in this study are specific to the MRI system used^47^. Additionally, the measurements were performed in a gel-filled phantom^12^,^13^,^31^,^47^. This approach implies lack of perfusion and does not take into consideration possible changes due to the thermoregulatory response in a patient^48^. Such a scenario is typically considered a worst-case, because perfusion can reduce significantly, e.g., two-fold, the heating of tissue in proximity of the lead^49^.

In this study, heating in the proximity of DBS implants, induced by the RF excitation pulses during the MRI, was measured by fluoroptic temperature sensors, which are the “state-of-the-art” in this field^31^,^47^,^50^,^51^,^52^,^53^. Another common approach for measuring temperature is MRI thermometry, which allows controlled heating while simultaneously measuring the spatial and temporal temperature distribution near the DBS implant. The most common MRI-thermometry method is based on proton resonance frequency shift (PRFS), which has been used to monitor temperature near a wire^54^. However, the susceptibility artifact from the DBS implant may extend up to 5 mm from the electrode surface, and at this distance the temperature changes are significantly lower than the peak temperature change^49^. Additional approaches were proposed to alleviate the susceptibility artifacts around a wire, but they did not provide real-time measurements at the desired high spatial resolution^55^,^56^, underestimating the peak temperature change. Conversely, fluoroptic thermometers can provide accurate and real time temperature measurements with a spatial resolution of typically 300 μm^53^.

The configuration of the implanted DBS components relative to the incident RF field and its orientation can also have a dramatic effect on the induced heating. In this study, the lead orientation was limited to a single case of overall lead length and path within the phantom^42^, i.e., lead placed parallel to the magnet bore axis (Fig. 1f). This allowed for the evaluation of the PtIr wire and the proposed RTS lead under the same conditions of high incident electric field (Fig. 1e) inside the homogenous phantom used in the study. However, the layout used does not necessarily model the exposure conditions of a lead implanted in a patient^57^, nor does it take into consideration differences between a single vs. bilateral lead. For example, a change in orientation of the lead with respect to a 1.5 T RF coil can generate changes in temperature of 20°C or more in a phantom^58^. A full systematic analysis of safety of the proposed RTS lead is still required and would need to include several configurations that would mimic clinically significant pathways, in line with the technical specifications proposed for safety analysis of patients with active implanted devices undergoing MRI^12^,^59^. Additional numerical simulations were performed with an electrically heterogeneous, anatomically precise human head and torso model^60^,^61^ containing a DBS lead reproducing a clinical case (Fig. 4b) — as shown by CT imaging (Fig. 4a) — for testing under different exposure and geometrical conditions. The model was placed with the head in the isocenter of an MRI coil (Fig. 4c). Figures 4f and 4g show a coronal and sagittal view the SAR in the head and body without an implanted lead, with a lead made of platinum iridium wire, and with an optimized wire-design RTS lead. These results also confirmed the advantage of the RTS design which significantly reduces absorbed power in the brain parenchymal near the electrode.

The benefits of the electrically thin design with its scattering behavior (see theoretical background on RTS design in Supplementary Information) can be used to replace any wire currently used in commercially available implant leads by coating a suture with biocompatible metals. Hence, a second more realistic and biocompatible RTS wire prototype (Thin-Films Research Inc., Westford MA) was created (Fig. 5a). Optical microscope (OM) images show the raw suture (Fig. 5b) before thin film deposition and after deposition (Fig. 5d). The abrupt transition of electrical conductivity between the two RTS layers (Fig. 5c) maximizes the mismatched impedance and the scattering within the fibers (Fig. 1 and Supplementary Information, theoretical background on RTS design). The two different layers of the RTS fiber and the surface characteristics were also studied with a Scanning Electron Microscope (SEM) (Fig. 5e and Fig. 5f) in order to better characterize the transition between the two layers.

The final RTS lead configuration is assembled similarly to the configuration of the Medtronic 3389 DBS lead (Fig. 4d) and thus it may be used (i.e., interchangeable) with the Medtronic Activa stimulator. Each lead consists of an implantable grade polyurethane inner shaft, into which the stylet is advanced, that terminates at the hermetically sealed distal tip of the lead. The RTS fibers are positioned around the inner shaft and contained within a protective sheath that has four platinum/iridium electrodes near the tip for delivery of stimulation to the target site. The proximal end of the lead also has four electrodes that interface with the implanted stimulation device after implantation. The leads are stereotactically introduced into the target and fixed at the skull with a burr hole cap and ring, as for the Medtronic DBS leads.

### Conclusions

This study presents a novel resistive-tapered stripline (RTS) lead design that “cloaks” the radiofrequency fields induced by magnetic resonance imaging (MRI) to reduce tissue heating, yet maintains the conduction of low-frequency stimulation from implanted medical devices. Computational modeling and simulations were used to find the optimal design parameters of the RTS lead. Polymer thick-film (PTF) technology was used to manufacture an initial prototype, which was tested in a 3 T MRI system showing a significant reduction of heating when compared to a Medtronic 3389 lead. Finally, state-of-the-art physical vapor deposition (PVD) technology was used to manufacture a biocompatible RTS wire prototype, which may easily replace any wire currently used in commercially available implant leads. The results shown suggest the proposed design may allow a significant increase in the number of patients with medical implants having safe access to the diagnostic benefits of MRI.

## Supplementary Material

Supplementary Information Supplementary Information

## Figures and Tables

**Table 1:** Uncertainty analysis. The methods used were based on the work of Neufeld et al.^42^. To evaluate the uncertainty of the quantities of interest derived by the simulations (i.e., 10 g-avg. SAR or the magnitude of incident electric field “E_RMS_”) Two simulations were run for each parameter by assigning two different values (“Val 1” and “Val 2”) to each parameter studied. The first value (“Val 1”) was the one used for the simulations shown in Fig. 2, whereas the modified value (“Val 2”) was set to a realistic value that could occur due to either design choice or manufacturing tolerance. The results obtained for each value (“Result 1” and “Result 2”, respectively) were used to evaluate sensitivity factor of the quantity evaluated (10 g-avg. SAR or magnitude of incident electric field “E_RMS_”). The standard deviation (“Std. Dev. ”) was derived from literature

Parameter	Quantity	Val 1	Val 2	Result 1	Result 2	Sensitivity Factor [%/mm]	Std. Dev	Uncertainty [%]
**Contact Width [mm]**	**10 g-avg. SAR at electrode [W/kg]**	0.381	0.762	6.98	7.22	9.12%	0.1	**0.9%**
**Contact Length [mm]**		1.5	3.0	6.98	7.28	2.85%	0.1	**0.3%**
**Contact Thickness [mm]**		0.0098	0.0196	6.98	7.00	32.6%	0.1	**3.3%**
**Substrate Thickness [mm]**		0.0254	0.0508	6.98	6.9	43.9%	0.1	**4.4%**
**Insulation Thickness [mm]**		0.0254	0.0508	6.98	7.07	52.0%	0.1	**5.2%**
**ε_r_ (Substrate)**		3.4	6.8	6.98	7.11	0.56%	2.00	**1.1%**
**ε_r_ (Insulation)**		2.5	5.0	6.98	7.00	0.13%	2.00	**0.3%**
**ε_r_ (Ink Lead)**		5.0	2.5	6.98	6.99	0.04%	2.00	**0.1%**
**σ (Contact) [S/m]**		9.3·10^6^	4.0·10^6^	6.98	6.99	0.00%	0.04	**0.0%**
**ε_r_ (Phantom)**		80	60	6.98	7.40	0.30%	2.00	**0.6%**
**σ (Phantom) [S/m]**		0.47	0.60	6.98	7.18	22.3%	0.04	**0.9%**
**Phantom Position X [mm]**	**E_RMS_, incident [V/m]**	0.0	10.0	300.9	302.2	0.04%	1.15	**0.1%**
**Phantom Position Y [mm]**		0.0	10.0	300.9	306.9	0.20%	1.15	**0.2%**
**Phantom Position Z [mm]**		0.0	10.0	300.9	307.0	0.20%	1.15	**0.2%**
**Lead Position X [mm]**		0.0	1.0	300.9	307.4	2.14%	0.58	**1.2%**
**Lead Position Y [mm]**		0.0	1.0	300.9	299.2	0.57%	0.58	**0.3%**
**Lead Position Z [mm]**		0.0	1.0	300.9	301.3	0.12%	0.58	**0.1%**
