# Supplementary material for: A Novel Brain Stimulation Technology Provides Compatibility with MRI
Source: Sci Rep. 2015 Apr 29;5:9805. doi: 10.1038/srep09805 (PMC4413880; doi:10.1038/srep09805)
Supplement: Supplementary Information [file srep09805-s1.pdf]

SUPPLEMENTARY INFORMATION

A Novel Brain Stimulation Technology provides compatibility with MRI

Peter Serano[a,b,c,1], Leonardo M. Angelone[c,1], Husam Katnani[d,e], Emad Eskandar[d,e], Giorgio Bonmassar[a,2]

[a] Athinoula A. Martinos Center, Massachusetts General Hospital, Charlestown, MA, USA

[b] Department of Electrical and Computer Engineering, University of Maryland, College Park, MD, USA

[c] Division of Biomedical Physics, Office of Science and Engineering Laboratories, Center for Devices and Radiological Health, U.S. Food and Drug Administration, Silver Spring, MD, USA

[d] Department of Neurosurgery, Massachusetts General Hospital, Boston, MA, USA

[e] Department of Neuroscience, Harvard Medical School, Cambridge, MA, USA

[1] P.S. and L.M.A contributed equally to this work.

[2] To whom correspondence should be addressed at: Athinoula A. Martinos Center, Massachusetts General Hospital, Harvard Medical School, Building 149, 13th Street, Charlestown, MA 02129, Tel. (617) 726-0962. Email: giorgio.bonmassar@mgh.harvard.edu

TABLE OF CONTENTS

SUPPLEMENTARY NOTES

Theoretical Background

Computational modeling and simulations

Lead design optimization

Additional analysis on effect of lead design

Temperature simulations

Uncertainty analysis

Bench test experiments

Temperature Measurements in MRI

IPG battery testing

SUPPLEMENTARY TABLE S1

SUPPLEMENTARY FIGURE S1

SUPPLEMENTARY FIGURE S2

SUPPLEMENTARY FIGURE S3

SUPPLEMENTARY REFERENCES

SUPPLEMENTARY NOTES

Theoretical background on RTS design

Resistively tapered Vee dipole antennas were first successfully introduced in landmine detection[1]. The design consisted of a linearly tapered thin film deposit, which allowed a radiation profile less affected by the ground properties. When the conductivity is tapered from the feed point to the extremity according to the Wu–King (WK) resistive profile[2], the antenna field radiation pattern uniformity from the feed point to the open end is greatly improved. Numerical electromagnetic (EM) simulations validated experimentally were successfully used in evaluating voltage differences within a 20% accuracy for an RTS design of thin triangular-shaped conductive sheets attached to a feeding transmission line by thin perfect electric conductor (PEC) traces[3]. In contrast with all previously proposed RTS designs, including the WK profile, which aimed to improve the antenna performance, the design proposed in this study decreases the antenna performance and the induced currents along the wire. The RTS implant exposed to an RF field can be represented with a hybrid model composed of an antenna attached to a transmission line, which consists of resistive traces with sharp changes in conductivity to maximize reflections, followed by a load such as an electrode connected to the tissue (Fig. 1a). As described in[4], the equivalent antenna (i.e., the entire RTS lead) receives the electromagnetic field and injects it into the first port (i.e., layer) with impedance Z_1 of such a network (Fig. 1b). A portion of the power transmitted to the first port of the RTS is reflected back as a result of an impedance mismatch between the first port and the antenna, while a remaining portion is supplied to the second layer of the RTS. The impedance of this second port is intentionally mismatched to reflect the greatest amount of power back to the implantable pulse generator (IPG) and away from the electrode that is in contact with the tissue. The fractional power reflected away and delivered to the tissue can be computed from the reflection Γ_2^{R} and transmission Γ_2^{T} coefficients:

$$\begin{aligned} \Gamma_0^R &= \frac{Z_1 - Z_0}{Z_1 + Z_0} & \Gamma_0^T &= \frac{2Z_1}{Z_1 + Z_0} \\ \Gamma_1^R &= \frac{Z_2 - Z_1}{Z_2 + Z_1} & \Gamma_1^T &= \frac{2Z_2}{Z_2 + Z_1} \\ \Gamma_2^R &= \frac{Z_L - Z_2}{Z_L + Z_2} & \Gamma_2^T &= \frac{2Z_L}{Z_L + Z_2} \end{aligned} \tag{S1}$$

One must consider the superposition of two steady state sine waves in the RTS traveling in opposite directions (Fig. 1b): one forward towards the tissue/electrode (blue) and one backward (red) reflected by the mismatched boundary towards the IPG. The first and second layer of the RTS act both as an antenna and transmission line for the signal that is reflected back away from the tissue/electrode. The overall signal Φ present in the electrode, which is the result of the superposition of all the direct and reflected back signals towards the first layer, is[4]:

$$\Phi = \Delta H_{OZ1}\,\sigma_1 L_1 + \Delta H_{OZ2}\,\sigma_2 L_2 - \left(\Gamma_0^R + \Gamma_1^R\right) - e^{-4(L_1\lambda_1 + L_2\lambda_2 + L_L\lambda_L)}\Big(\Gamma_1^R e^{2(L_1\lambda_1 + 2L_2\lambda_2 + 2L_L\lambda_L)} + \Gamma_2^R e^{2(L_1\lambda_1 + L_2\lambda_2 + 2L_L\lambda_L)} + \Gamma_2^R e^{2(2L_1\lambda_1 + L_2\lambda_2 + 2L_L\lambda_L)} - \lambda_L^2 e^{-4L_1\lambda_1}\left(\Gamma_0^R + \Gamma_1^R\right) - \lambda_L^2\left(\Gamma_0^R + \Gamma_1^R + \Gamma_2^R\right)\Big) - \Gamma_L^R\left(e^{-2(L_1\lambda_1 + L_2\lambda_2 + L_L\lambda_L)} - \lambda_L^2 e^{-2(2L_1\lambda_1 + L_2\lambda_2 + L_L\lambda_L)}\right) \quad \text{(S2)}$$

where λ_i is the propagation coefficient, L_1 and L_2 are the lengths of the first and second layer, respectively, $\Delta H_{OZ1}\sigma_1 L_1$ is the signal received by the first layer of the RTS, and $\Delta H_{OZ2}\sigma_2 L_2$ is the signal received by the second layer. The signal Φ that emerges from the left side of the port is the sum of all the terms that represent the intrinsic reflections in the RTS. The terms ΔH_{OZ1}, ΔH_{OZ2} (i.e., the variation of the magnetic field along the RTS or *z*-direction in the two adjacent Yee cells), and λ_i can be estimated using a numerical approach, such as the finite element method (FEM) algorithm used herein, based on the particular geometrical model considered[5].

Equation (S2) has four positive definite unknowns, i.e., σ_1,σ_2, L_1, andL_2. Furthermore, there are two additional minimization constraints on the upper limit (σ_T) of the desired overall RTS resistance and total RTS length:

$$0 > \sigma_1 + \sigma_2 \geq \sigma_T \quad , \quad L_1 + L_2 = L \quad \text{(S3)}$$

Equation (S2) has been introduced only to give an insight into the theory of RTS design. The following section presents a more detailed description of the fields inside the RTS leads.

The peak inductance of the lead can be estimated as follows:

$$\boldsymbol{L_{MAX}} = \boldsymbol{\mu} \frac{\iiint_{\Re} \|\boldsymbol{H}(x,y,z)\|^2 \, dx \, dy \, dz}{\left(\iint_{\theta_{1,2}} \boldsymbol{J}(x,y,z) \cdot \boldsymbol{k}(x,y,z) \, dS\right)^2} \tag{S4}$$

where $\|\boldsymbol{H}(x, y, z)\|$ is the complex magnitude of the Fourier transform or harmonic component of the magnetic field at the Larmor frequency f_0 = 128 MHz, $\boldsymbol{\mu}$ is the permeability of the material (see Supplementary Table S1), $\Re$ is the domain composed of RTS wire and insulation, $\theta_{1,2}$ is the section, k is the unitary vector between layers 1 and 2 of the RTS, and $\boldsymbol{J}(x, y, z)$ is the current density inside the lead. One can only estimate $\boldsymbol{L_{MAX}}$ or the peak inductance of an ideal inductor which is an ideal magnetic field generator that stores the magnetic field energy generated by the supplied current, whereas in a real inductor the inductance L is always lower than $\boldsymbol{L_{MAX}}$ because of magnetic flux losses.

The main or static magnetic field B_0 present in an MRI will produce a spin or precession of nuclei of the hydrogen atoms (protons) in the water molecules in the tissue. The precessional path of these protons around the magnetic field is circular like and sometimes described in terms of a spinning top. The Larmor or precessional frequency in MRI refers to the rate of precession of the magnetic moment of the proton around the external magnetic field and is related to the strength of the magnetic field B_0. The frequency of all fields considered here is the Larmor frequency (i.e., 128 MHz at 3T). The behavior of a RTS wire inside an electromagnetic field can be studied as a linear antenna under the thin wire assumption (i.e., the diameter d of the geometry is $d < \lambda/100$, i.e. d=100 μm). For an ideal linear thin antenna the current density $\boldsymbol{J}(x, y, z)$, which determines the fundamental fields $\mathbf{H}(x, y, z)$ and $\mathbf{E}(x, y, z)$ is[6]:

$$\boldsymbol{J}(x,y,z) = \boldsymbol{k}I(z)\delta(x)\delta(y) \tag{S5}$$

where $\boldsymbol{k}$ and $I(z)$ are the unit vector and current intensity along the implant along the z-axis, as shown in Fig. 1c. The current density field can be found by solving the following Pocklington's integral equation[7]:

$$\int_{-L/2}^{L/2} I(z)\left(\frac{\partial^2}{\partial z^2} + k^{\,2}\right) G(z-z')dz' = -j\omega\varepsilon_w(z)\left\|\boldsymbol{E}_{\boldsymbol{B}_1}(z)\right\| + I(z)\frac{j\omega\varepsilon_w(z)}{\sigma_w(z)} \tag{S6}$$

where $\boldsymbol{E}_{\boldsymbol{B}_1}(z)$ is the electric field on the surface of the wire induced by the $\mathbf{B_1}$ field, the constant $k = 2\pi/\lambda$ is the free space wave number, $\varepsilon_w(z)$ and $\sigma_w(z)$ are the electrical permittivity and conductivity, respectively, along the wire, and $G(z)$ is Green's function. Note that equation (S6) also includes the conductivity profile of the wire, which is not typically present in implanted wires due to the common assumption of an ideal conductor. The kernel that approximates the exact Green's function of the integral in equation (S6) is given the following[6]:

$$G(z-z') = \frac{e^{-jkR}}{R}, R = \sqrt{(z-z')^2 + \frac{d^2}{4}} \tag{S7}$$

In the case of the RTS geometry, equation (S6) becomes:

$$\int_{0}^{L_2} I(z)\left(\frac{\partial^2}{\partial z^2} + k^2\right)\frac{e^{-jkR}}{R}dz' + \int_{L_2}^{L_1+L_2} I(z)\left(\frac{\partial^2}{\partial z^2} + k^2\right)\frac{e^{-jkR}}{R}dz' = -j\omega\varepsilon_w(z)\left(\left\|\boldsymbol{E}_{\boldsymbol{B}_1}(\boldsymbol{z})\right\| - \frac{I(z)}{L_w(z)\sigma_w(z)}\right) \tag{S8}$$

where $\mathrm{\sigma_w(z), \varepsilon_w(z)}$ and $\mathrm{L_w(z)}$ are respectively equal to $\mathrm{\sigma_1, \varepsilon_1}$ and $\mathrm{L_1}$ when $\mathrm{L_2 \geq z \geq L_1 + L_2}$ and equal to $\mathrm{\sigma_2, \varepsilon_2}$ and $\mathrm{L_2}$ when $\mathrm{z < L_2}$.

The following current distribution along the wire is an approximate solution of equation (S8):

$$I(z) = \begin{cases} I_2 sin\big(k(L_1 + L_2 - z)\big) & L_2 \geq z \geq L_1 + L_2 \\ I_1 kz & z < L_2 \end{cases} \quad \text{(S9)}$$

Equation (S9) is the typical shape of the ideal current distribution in an RTS wire as sketched in Fig. 1c. A more precise current distribution estimated using EM numerical simulations is shown in Fig. 2d. The RTS design reduces the overall inductance of the lead (Fig. 2c). Additionally, the current density has a minimum value along the lead in proximity to the electrode (Fig. 2d), thereby reducing the risk for energy absorption in the surrounding tissue.

Finally, the skin depth δ_s plays an important role in the RTS design:

$$\delta_s = \sqrt{\frac{2}{\sigma\mu\omega}} \quad \text{(S10)}$$

where $\omega = 2\pi f = 2\pi \cdot 128 \cdot 10^6\ Hz$, $\mu = 4\pi \cdot 10^{-7}\ H/m$, and $\sigma_1 = 1.968 \cdot 10^6\ S/m$ $\sigma_2 = 25.61 \cdot 10^3\ S/m$ are the conductivity of the two RTS layers, respectively, calculated by the simulations. Note that for these values, the skin depth of the optimal RTS design was 31.63 μm for the first layer and 278 μm for the second layer, which is higher than the thickness of the modeled lead, which was 9.7 μm. The FEM simulation results shown in Fig. 2 take in consideration the skin depth effect.

Computational modeling and simulations

Lead design optimization - All lead design optimization included models of a realistic MRI birdcage transmit coil tuned at the Larmor Frequency f_0=128 MHz, an ASTM phantom [8,9], a PtIr wire, and a realistic RTS lead that allowed for a physically realizable solution. The dimensions and material properties of the coil, lead, and phantom are listed in the Table S1. Detailed methods on the simulations can be found in[10]. The computational models were created with the

finite element methods (FEM) electromagnetic solver high frequency structure simulator (HFSS) v15.0 and circuit solver Designer v8.0, (ANSYS, Inc., Canonsburg, PA). The FEM method[11] allowed for high geometrical modeling accuracy (i.e., minimal tetrahedral length equal to 45 μm) at the electrode, where the highest electric field was observed[12-14]. The parameter used to evaluate numerically the energy deposition in the phantom was the specific absorption rate (SAR) averaged in a volume with a 10g mass (10g-avg. SAR) [15]. SAR (W/kg) is a measure of the energy rate absorbed by the human body when exposed to a RF field and is the dosimetric parameter used in RF safety guidelines. For each lead design simulation, the 10g-avg. SAR was computed in a location at 0.1 mm from the anterior face of the lead contact in the direction of the positive Z-axis. In order to obtain a high increase of 10g-avg. SAR, the lead was placed in a volume with high tangential electric field magnitude[16] (see Fig.1e).

The flat-design RTS lead (Fig. 3a) contained two discrete sections of variable conductivity and length, connected in series. Three requirements were used to minimize the optimal design search including: a) total length fixed to 40 cm to match common lead lengths for implantable devices[17], b) conductivity of the proximal section higher than the distal section; and c) total low-frequency resistance of the lead equal to 400 Ω, i.e., less than the typical impedance in patients[18]. Additional numerical simulations were also performed in order to determine the best design parameters and estimate the performance of the wire-based RTS design since it utilizes different materials and geometry. A detailed view of this model can be seen in Fig. 4d. The model contains four identical RTS fibers each divided into six fixed-length sections of variable thickness which allow the model to simulate the effects of varying length (by making two or more adjacent sections equal in thickness) and number of sections (by choosing the number of changes in adjacent section thickness). Total lead resistance was still R = 400 Ω and the ratio of

layer thicknesses $t_1/t_2 = 20$. The simulation showed that the optimal design was obtained by dividing the lead into two sections of equal lengths $L_1 = 0.2$ m and $L_2 = 0.2$ m and yielded a 33% reduction in peak 10g-avg. SAR within 1cm of the lead (see also Fig. S1 for electric field and magnetic field maps). A summary of optimal designs by number of sections can be seen in Fig. 4e.

Additional analysis on effect of lead design - Additional simulations were performed to evaluate the effect on 10g-avg. SAR of different design variables, including: shape (i.e., wire vs. thin), lead conductivity, and proximal end boundary conditions (i.e., insulated vs. uninsulated). The results of the simulations are shown in the Supplementary Fig. S2. Notably, the simulations, manufacturing, and bench testing were performed with the electrode exposed only on a single side, although additional simulations included the case of two exposed ends. Higher SAR was predicted for models with wires compared to thin RTS geometries, in line with the skin depth calculations discussed in equation (S10). Furthermore, based on the selected design the RTS leads showed lower SAR than leads with homogeneous conductivity (see also Fig. 2a, case $\sigma_1/\sigma_2=1$).

Temperature simulations - The temperature simulations were performed by implementing the following heat equation in solids, which corresponds to the differential form of Fourier's law:

$$\rho C_p \frac{\partial T}{\partial t} = \nabla \cdot (k_T \nabla T) + \iiint_{\emptyset} \frac{\sigma \|E\|^2}{\rho} \, dx \, dy \, dz \qquad \text{(S11)}$$

where T [K] is the absolute temperature, C_p [J/(kg K)] is the specific heat capacity at one atmosphere of constant pressure, ρ [kg/m^3] is the mass density, k_T [W/(m K)] is the thermal conductivity (see Table S1 for values) and $\emptyset$ is the volume of 10 g of polyacrylic acid (PAA) in the point where T is estimated. The temperature distribution values of T were estimated by

solving the heat equation in solids (equation S11), which is used to model heat transfer by conduction only. The equation was solved considering as a heat source term the SAR calculated by the HFSS-based EM simulations (right term). The geometry consisted of two blocks modeling the ASTM phantom (410×80×590 mm^3 and 150×80×290 mm^3). The external surfaces of the ASTM phantom were set to Dirichlet boundary conditions with $T = 20.15^{o}$C. The solution was calculated in a Cartesian 3D coordinate system and consisted of temperature values T. The calculations were performed using a FEM-based commercially available software (Multiphysics 4.4, COMSOL, Burlington, MA).

Uncertainty analysis

A simulation study to assess the uncertainty of design and simulation parameters was performed (Table 2). The parameters studied were selected such that they could be considered independent. The methods used were based on the work of Neufeld et al.[13]. To determine the impact of the contribution of an individual parameter to the total uncertainty of the simulations, first two simulations were run for each parameter by assigning two different values ("Val1" and "Val2" in Table S2) to each parameter studied. The first value ("Val1") was the one used for the simulations shown in Fig. 2 whereas the modified value ("Val2") was set to a realistic value that could occur due to either design choice or manufacturing tolerance. Assuming linear dependence of the measurement values on the varying parameter, a sensitivity factor was determined for each parameter by calculating the percent error difference between the two evaluation results and then dividing by the absolute value of the change in parameter value. The individual uncertainty contribution was then calculated by multiplying the sensitivity and the standard deviation of the parameter uncertainty. The standard deviations were small for parameters such as the implant length, which can be accurately determined, and large for parameters such as the conductivity.

The analysis confirmed a high sensitivity – and relative high uncertainty – to the thickness and dispersion properties of the insulation layer, in line with previous studies[8]. Lower SAR values resulted with a 25.4 μm vs. 50.8 μm dielectric thickness of both insulation and substrate, which is consistent with the notion that insulation characteristics strongly affect the antenna behavior[12]. Moreover, Fig. 2a shows that the ideal length of layer 2 is 3.3 cm, thus microscopic surface mount resistors (e.g., $0.4 \times 0.2 \times 0.2$ mm^3), often connected to each electrode in commercial EEG/fMRI caps, and cannot be used to create an ideal RTS geometry since they are too short. Additionally, larger resistors would be too bulky and rigid to be attached to a microscopic wire of an implant. Finally, the simulations showed that conductor thickness plays a fundamental role in the RF-induced currents[19], with a low uncertainty coefficient (see Table S2). Notably, a homogenously conductive thin design decreased the current density at thicknesses less than the skin depth (Supplementary Fig. S2). The uncertainty analysis performed showed that the permittivity of the binder used in conductive inks can significantly affect 10g-avg SAR. Binders[20] serve to bind together the nanoparticles of the material, ensure the necessary viscosity for proper transfer of the ink from the press to the substrate, provide adhesion to the substrate, and contribute to the drying speed and resistance properties of the ink[21]. The relative permittivity of binders varies from two to fifteen[22] or higher in composites[23], and it is essential for the RTS effect presented in this paper, as no RTS effect was found in simulations with binders with the unity relative permittivity of vacuum.

Bench test experiments

Manufacturing of flat-design RTS prototype - A flat-design RTS lead prototype was manufactured using two different commercially available conductive ink materials for initial proof-of-concept testing. The first ink (479SS, Electrodag, Acheson LTD., Kitano, Japan) is a

silver (Ag) based PTF ink and was fixed to a specified resistivity of 0.02 Ω/sq./mil[24]. This ink was used in the higher conductivity section L_1. The second ink (423, Electrodag) is a carbon (C) based PTF ink, which has significantly higher resistivity compared with Ag based PTF inks of 42 Ω/sq./mil[25]. The final layer L_2 was fabricated by chemically mixing the two PTF inks to adjust the conductivity of the second section to the value prescribed by the simulations, which was constrained to be within the conductivity values of the Ag and C-based PTF inks. The length of layer 1 was proportional to the conductivity of the silver ink and was fixed to allow a target ratio $\sigma_1/\sigma_2 = 77$ with the optimal total resistance of 400 Ω. Notably, the resistivity of both the carbon traces and silver electrodes is constant from 100 Hz to 200 MHz[21]. The 400 Ω resistance was well within the range of current commercial IPGs considering that the contact electrode/tissue resistance is usually below 1.5 kΩ[18]. The tolerance for resistivity was 5% and the tolerance for length was 50 μm.

Temperature Measurements in MRI - Evaluation of RF-induced heating during MRI was assessed by loading the MRI RF body coil with a standard ASTM phantom [9] (Fig. 3b). The phantom shell was made of Plexiglas and filled to a volume of 24.6 L with a gel consisting of PAA (436364, Sigma Aldrich Co., St. Louis, MO), distilled water (conductivity less than 10^{-3} S/m), and reagent grade NaCl (S9888, Sigma Aldrich Co., St. Louis, MO). The ratio of the mixture was 1.32 g NaCl and 10 g PAA for each 1 L of water to obtain a conductivity of approximately 0.47 S/m at room temperature [9]. The mixture created a semisolid gel that approximated the dielectric constant and thermal convection of human tissue[26]. A plastic scaffold with adjustable posts (i.e., plastic screws, bolts and washers) was placed on the far right side of the phantom and was utilized to consistently position system components (i.e., electrode lead and temperature probes) within the phantom (Figs. 3c and 3d). As shown in Fig. 1e, the electric field

magnitude inside the phantom at 128 MHz in the selected configuration was highest in a location close to the body coil[13]. In order to have high magnitude of electric field incident to the lead, the phantom was shifted as far right as possible within the bore and the leads were placed on the side of the phantom (Fig. 3b, Fig. 1e and 1f). Temperature measurements were recorded using four fiber-optic temperature sensors compatible with MRI (Neoptix Reflex, Qualitrol LLC, Fairport, NY). Three thermometry probes were positioned within the phantom to capture the temperature profiles of set points on the electrode lead. Probes were positioned (Figs. 3c and 3d) perpendicular to the electrode contact (0.3 mm from the center of the contact), in-line with the tip (0.3 mm away from tip), and perpendicular to the middle of the lead (0.3 mm from center of lead)[27]. The 3T MR system (Skyra, Siemens Medical Systems, Erlangen, Germany) used for imaging was programmed with parameters set to investigate high RF energy exposures that meet the maximum limit allowed for averaged SAR generated by an MRI scan (Turbo Spin-echo sequence, TR=654 ms; TE=16 ms; 180° flip angle; $1.2\times1.2\times1.2$ mm^3 voxel volume). Each experimental session consisted of a 5-minute epoch for baseline temperature recordings, a 15-minute MRI scan, and a 15-minute epoch post-scan to assess temperature decay rates. A commercially available DBS lead (3389, Medtronic Inc., Minneapolis, MN) and the RTS prototype were tested in the same sessions. The Medtronic 3389 lead was chosen because it is approved for use in MRI with very specific conditions[28]. The proximal end of the Medtronic 3389 lead was not insulated. Temperature was recorded at 1-second intervals. A baseline experimental session in which no lead was positioned within the scaffolding of the phantom was conducted to establish the RF-induced heating of the phantom alone.

The design optimization was performed with a model of a body coil based on literature[29]. In order to compare the temperature measurements with the simulations, additional simulations

were performed with a second body coil model, matching the geometrical characteristics of the coil used in the MRI measurements (i.e., 70 cm diameter, 40 cm length, tuned to a Larmor frequency of 123.2 MHz). The coil was loaded with the ASTM phantom placed off-center, as in the experimental setup (Fig. 3b). Supplementary Figure S3 shows the results of the temperature simulations compared with the experimental measurements (shown also in Fig. 3e).

Implantable pulse generator (IPG) battery testing - The testing was performed using the following parameter settings for the Activa PC IPG (Medtronic, Inc., Minneapolis, MN): i) single lead (contact 0, according to the Medtronic numbering in Fig. 4d) set to negative (-), ii) case set to positive (+ and contacts 1, 2 and 3 were all turned off), iii) unipolar cathodic pulse train set to an amplitude of 2 V, a frequency of 130 Hz and a pulse width of 90 μs. For the Medtronic 3389 lead the following procedure was used: the extension wire was connected and fastened (with set screw) on one side to the IPG and on the other side to the lead. The connection site was covered with silicon wrap and sealed on both ends with non-dissolvable suture, which is the same procedure utilized in the operating room to produce a water tight implant. For the RTS lead the following procedure was used: The extension wire was connected and fastened (with set screw) to the IPG and the RTS lead was connected (contact 0) to the extension wire with silver epoxy (8331, MG Chemicals, Surrey B.C., Canada) and insulated with super glue. The connection site was covered with silicon wrap and sealed on both ends with non-dissolvable suture. A digital multimeter was used to check for conductivity between RTS contact and extension wire. For both leads, the IPG, extension, and lead were placed in a quart of deionized water. Saline solution at room temperature was added to the deionized water until the impedance measured 1500 Ω. Because the lead impedance of the RTS prototype was higher than the Medtronic 3389 lead, a lower amount of saline solution was added for the latter case. A hand-

held Medtronic Physician programmer was utilized to activate the IPG to test for therapeutic impedance. As the proprietary battery and circuit designs of the IPG were unknown, a direct quantification of power consumption could not be calculated. Therefore, battery level (in volts, analogous to the indicator on a cell phone or laptop) was measured using the handheld programmer. The battery level provides an indication of power consumption. Although the exact relationship between battery level and power consumption is not disclosed by the manufacturer, the changes in battery level recorded can still inform about possible changes in the amount of charge delivered when comparing the two lead designs. The IPG was turned on and left on for a total of four weeks; during this time the IPG was tested once a week by two measures, 1) an oscilloscope was used to check and measure the emitted pulse train in saline solution, and 2) the hand-held programmer was used to monitor changes in the therapeutic impedance. Saline was added to the water if impedance dropped below 1500 Ω (Figs. 4f and 4g). Testing was done with constant impedance to check that — with given conditions — the RTS would deliver the same charge compared to the Medtronic 3389. When implanted, the impedance of the RTS (~400 Ω) would in fact be different from the Medtronic 3389 (~50 Ω), which can result in a lower battery life or a shorter time period before recharging[30]. However, any overall differences in total impedance would still be within the normal variability (between 500Ω and 1.5 kΩ) measured in patients a few weeks post-implantation[18].

Manufacturing of wire-design RTS prototype - A second RTS wire prototype was manufactured using the state-of-the-art physical E-Beam vapor deposition (PVD) technology (Thin-Films Research, Inc., Westford, MA) over a rotating suture (Ethilon 6-0, Ethicon Inc., Bridgewater, NJ) substrate (80 µm diameter), which was selected for its biocompatibility. The polymer fiber core was composed of Ethilon 6.0 (Ethicon Endo-Surgery Inc., Somerville, NJ), a

non-absorbable monofilament suture composed of the long-chain aliphatic polymers Nylon 6 and Nylon 6.6, due to its mechanical properties (e.g., tensile strength) and long history of use as a permanent implantable material[31]. Ethilon has excellent mechanical properties, for instance the selected 6-0 fiber size or 80μm has a maximum tensile strength of 6.68N[32], which diminished by only 10% after thin film deposition. Ethilon has also a very high breaking stress of 6.25GDP compared to other non-absorbable sutures[33], which may increase long term mechanical stability and reduce likelihood of failures. The Ethilon nylon core was coated first with titanium and then with gold, using PVD. The suture was first coated with a thin Ti layer (100 nm) to improve bonding between gold and the polymer core fiber[32,34]. Subsequently the two-layers of RTS microwire were formed by depositing gold layers of two different thicknesses, one for each layer (i.e., 600nm layer 1 and 1,000nm layer 2) to allow the creation of a different conductivity within each layer. The two thickness levels were selected in order for the two layers to match the conductivity prescribed by the simulations (Fig. 4). The gold coating produced the desired conductivity profiles while maintaining the proper resistivity and inductance properties. The RTS core was finally coated with Parylene and marked with a biocompatible ink to indicate the direction in which the core is to be connected to the electrodes and the proximal connector. Parylene C was used for the conductor wire insulation since it can be applied at room temperature which avoids exposure of the RTS core wire to high temperatures during processing. Medical grade Parylene C is an excellent neural prosthetic coating as shown by the good encapsulation overall performance[35]. The RTS wire will be inserted in the original Medtronic 3389 design, which has a stylet in the lumen which is removed during surgery that confers the implant the rigidity to reach the target. In regards to the long-term implantation, following the

Medtronic 3389 design, the RTS wire will be completely encapsulated in biocompatible and implantable polymers that enhance durability.

Geometry	Dimension
Coil Diameter	610 mm*
Coil Length	620 mm*
Coil Shield Diameter	660 mm*
Coil Shield Length	1220 mm*
Coil and Shield Thickness	0.1 mm*
Coil Ring/Rung Width	25 mm*
Coil Former Inner Diameter	590 mm*
Coil Former Wall Thickness	10 mm*
Lead Length	40 cm
Lead Width	0.5 mm
Lead Thickness	15.7 µm
Contact Length	1.5 mm
Contact Width	0.5 mm
Lead Substrate Width	10 mm
Lead Substrate Thickness	25 µm
Lead Insulation Width	5 mm
Lead Insulation Thickness	25 µm
**Material**	**Value**
Copper (Coil and Shield) Conductivity	$5.8\ 10^{7}$ S/m
PMMA (Coil Former) Permittivity	3.0
Platinum (RTS Contact) Conductivity	$9.3\ 10^{6}$ S/m
PtIr (Electrode/Wire) Conductivity	$4.0 \cdot 10^{6}$ S/m
ASTM Phantom Conductivity	0.47 S/m**
ASTM Phantom Permittivity	80**
Kapton HN (Lead Substrate) Permittivity	3.5
DI-7502 (Lead Insulation) Permittivity	2.5
Conductive Ink (Lead) Permittivity	5.0
PAA Gel (ASTM Phantom) Thermal Diffusivity	$1.3 \cdot 10^{-7}$ m/s^{2}**
PAA Gel (ASTM Phantom) Specific Heat Capacity	4150 J/(kg·C)**
PAA Gel (ASTM Phantom) Density	1200 kg/m^{3}**

Supplementary Table S1 (Top) Dimensions of numerical model. Coil dimensions (indicated with *) were based on the work of Yeo et al. [29] (Bottom) Electrical and thermal properties used for the simulations. The properties of the ASTM phantom (indicated with **) were based on the ASTM standard [9].

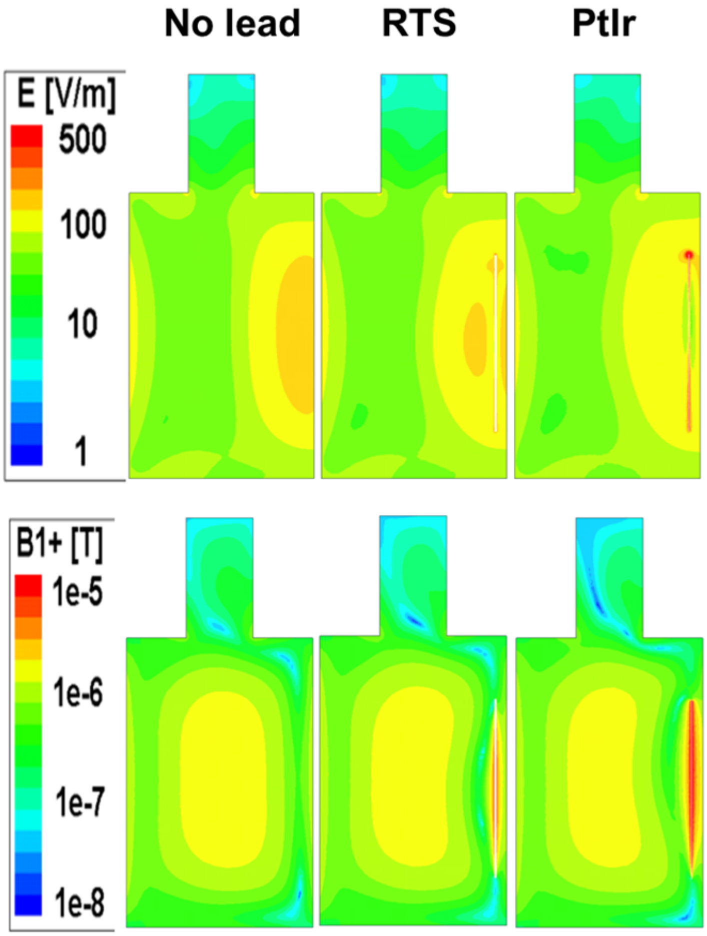

Supplementary Figure S1 | Electromagnetic simulations. Maps of (top) electric field and (bottom) B_1^+ field magnitude in the coronal (XZ) plane of the ASTM phantom at the isocenter of the lead. The B_1^+ field is the circularly polarized component of the magnetic field used to elicit the MRI signal_ENREF_37[36]. Three cases are presented: (1st column from the left) phantom model without lead, (2nd column) with the selected RTS lead (i.e., L_1=0.367 m, L_2 = 0.033 m, σ_1=1.968·10^6 S/m, and σ_2=25.61·10^3 S/m), and (3rd column) with a PtIr wire (100 µm diameter, 40 cm long). The top row shows the magnitude of the electric field at the computed frequency. The electric field peaks near the electrode and was maximum for the PtIr lead (3rd column), whereas it was much lower with the RTS (2nd column) and the ASTM phantom without lead (1st column). The second row shows the magnitude of B_1^+ field. The ASTM phantom without lead is the reference and any change in B_1^+ field near the lead when introducing the implant into the phantom with respect to the reference implies distortions in the field homogeneity.

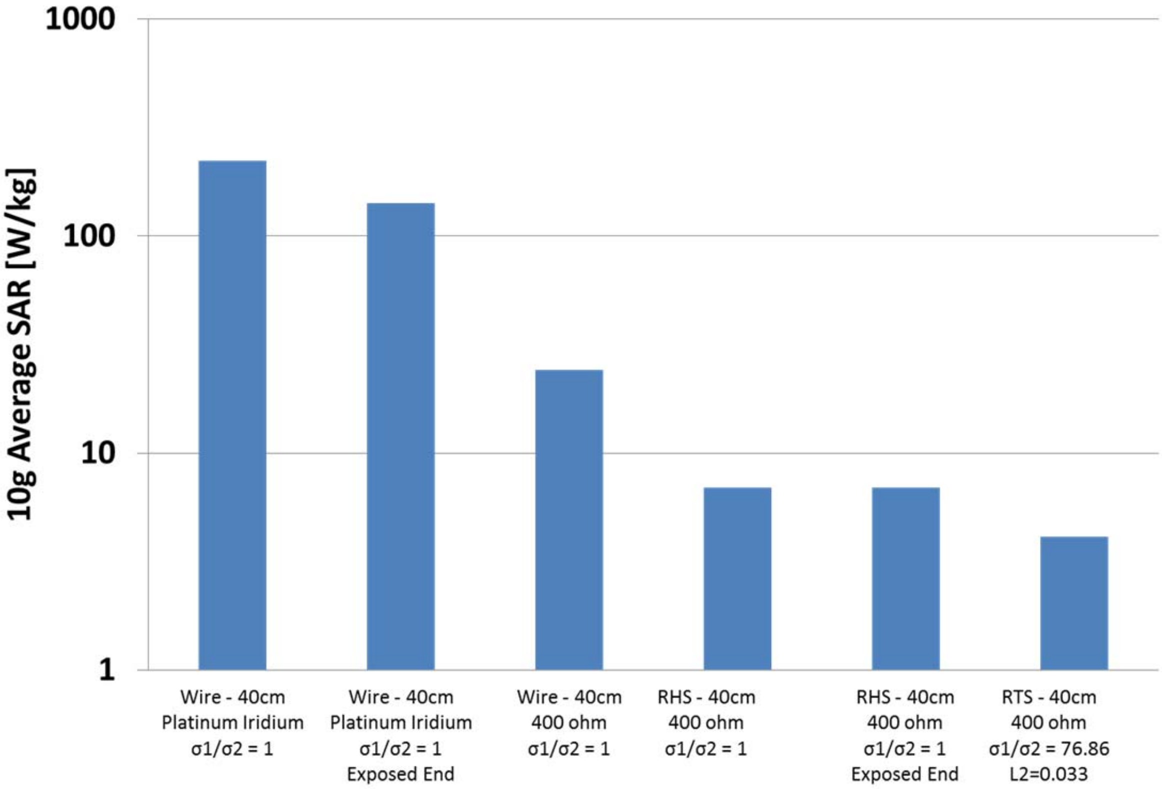

Supplementary Figure S2 | Effect of lead configuration on 10g-avg. SAR. Histogram showing 10g-avg. SAR in the phantom at 0.1 mm from the electrode calculated with different lead configurations. The 10g-avg. SAR varied with shape (i.e., wire vs. thin) and conductivity (homogeneous vs. RTS). These results illustrate that — with the configurations evaluated — wires produce larger peak SAR than thin leads. Additionally, the RTS design allowed for lower peak SAR when compared with a resistively homogeneous (i.e., σ_1/σ_2=1) thin stripline.

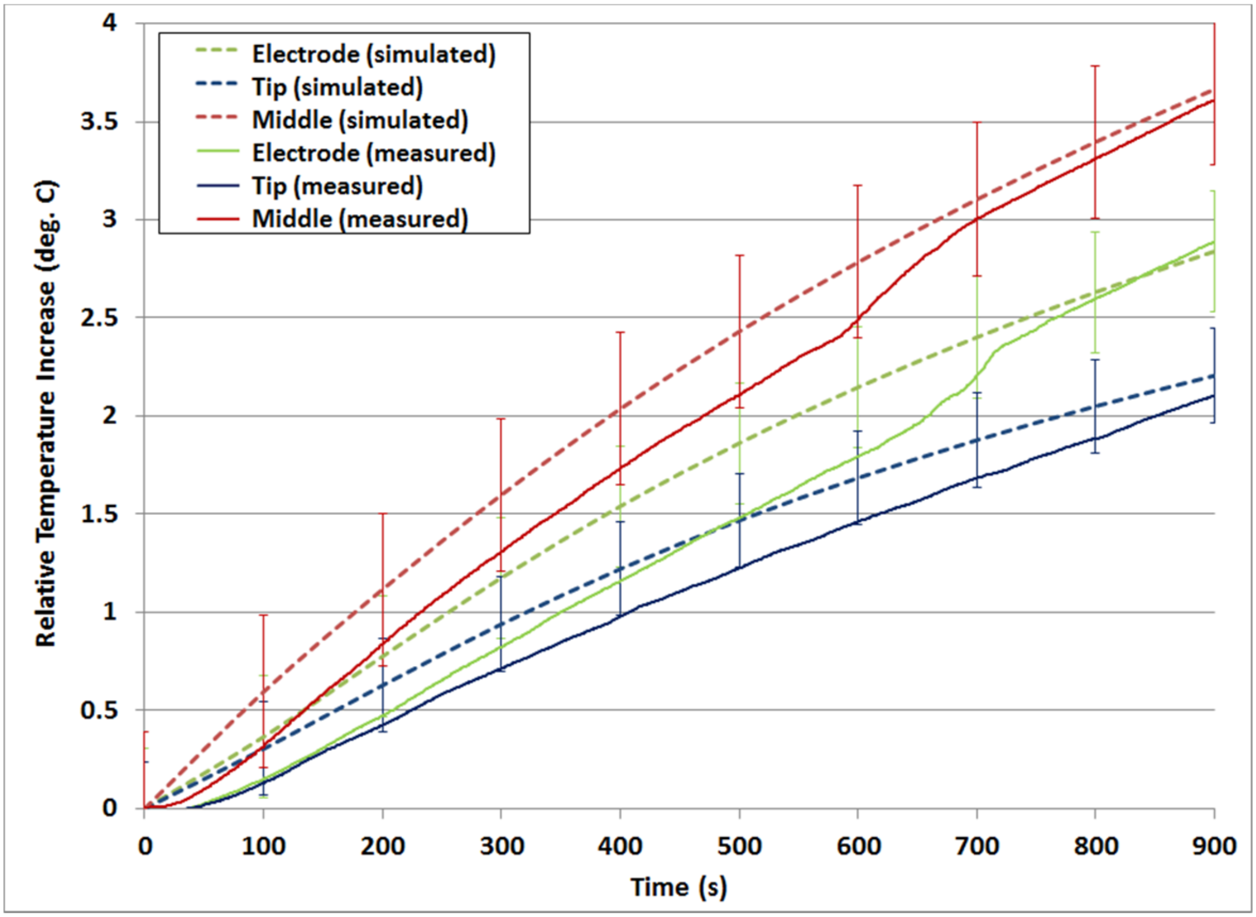

Supplementary Figure S3 | Temperature measurements in MRI vs. numerical simulations. Temperature simulations with standard error as computed by the model used for the RTS design compared with the temperature changes measured in the MRI scanner. Simulations and measurements agreed within the standard error.

SUPPLEMENTARY REFERENCES

1 Kanda, M. A relatively short cylindrical broadband antenna with tapered resistive loading for picosecond pulse measurements. *IEEE Trans Antennas Propag* **AP-26** (1978).

2 Wu, T. T. & King, R. The cylindrical antenna with nonreflecting resistive loading. *IEEE Trans Antennas Propag* **AP-13**, 369-373 (1965).

3 Taflove, A. & Hagness, S. C. *Computational electrodynamics : the finite-difference time-domain method*. 2nd / edn, (Artech House, 2000).

4 Bonmassar, G. Resistive Tapered Stripline (RTS) in Electroencephalogram Recordings During MRI. *IEEE Trans on Microw Theory and Tech* **52**, 1992-1998 (2004).

5 Jin, J.-M. *The finite element method in electromagnetics*. (Wiley, 1993).

6 Orfanidis, J. *Electromagnetic Waves and Antennas, www.ece.rutgers.edu/~orfanidi/ewa*. (2013).

7 Levy, M. *Parabolic equation methods for electromagnetic wave propagation*. (Institution of Electrical Engineers, 2000).

8 Bassen, H., Kainz, W., Mendoza, G. & Kellom, T. MRI-induced heating of selected thin wire metallic implants-- laboratory and computational studies-- findings and new questions raised. *Minimally invasive therapy & allied technologies : MITAT : official journal of the Society for Minimally Invasive Therapy* **15**, 76-84, doi:10.1080/13645700600640931 (2006).

9 ASTM-F2182-11. *Standard Test Method for Measurement of Radio Frequency Induced Heating On or Near Passive Implants During Magnetic Resonance Imaging*, <http://www.astm.org/Standards/F2182.htm> (2011 - Date of access: 27/01/2015).

10 Bonmassar, G., Serano, P. & Angelone, L. M. Specific absorption rate in a standard phantom containing a Deep Brain Stimulation lead at 3 Tesla MRI. *6th International IEEE/EMBS Conference on Neural Engineering (NER)*, 747-750, doi:10.1109/ner.2013.6696042 (2013).

11 Kozlov, M. & Turner, R. Fast MRI coil analysis based on 3-D electromagnetic and RF circuit co-simulation. *Journal of magnetic resonance* **200**, 147-152, doi:10.1016/j.jmr.2009.06.005 (2009).

12 Guy, A. Biophysics-energy absorption and distribution. *AGARD Lecture Series, Radiation Hazards (Non-ionizing Radiations--Biological Effects and Safety Considerations* **78** (1975).

13 Neufeld, E., Kuhn, S., Szekely, G. & Kuster, N. Measurement, simulation and uncertainty assessment of implant heating during MRI. *Physics in medicine and biology* **54**, 4151-4169, doi:10.1088/0031-9155/54/13/012 (2009).

14 Mohsin, S. A., Sheikh, N. M. & Saeed, U. MRI-induced heating of deep brain stimulation leads. *Physics in medicine and biology* **53**, 5745-5756, doi:10.1088/0031-9155/53/20/012 (2008).

15 IEC-60601-2-33. *International Electrotechnical Commission - International Standard, medical equipment - part 2-33: Particular requirements for the safety of the magnetic resonance equipment for medical diagnosis, 3rd edition*, 2010).

16 Jin, J.-M. *Electromagnetic analysis and design in magnetic resonance imaging.* (CRC Press, 1999).

17 Rezai, A. R. *et al.* Neurostimulation systems for deep brain stimulation: in vitro evaluation of magnetic resonance imaging-related heating at 1.5 tesla. *Journal of magnetic resonance imaging : JMRI* **15**, 241-250 (2002).

18 Volkmann, J., Herzog, J., Kopper, F. & Deuschl, G. Introduction to the programming of deep brain stimulators. *Movement disorders : official journal of the Movement Disorder Society* **17 Suppl 3**, S181-187 (2002).

19 Merilampi, S. L. *et al.* The Effect of Conductive Ink Layer Thickness on the Functioning of Printed UHF RFID Antennas. *Proceedings of the IEEE* **98**, 1610-1619, doi:10.1109/jproc.2010.2050570 (2010).

20 Osborne, I., Lavine, M. & Coontz, R. Materials for electronics. Looking beyond silicon. Introduction. *Science* **327**, 1595, doi:10.1126/science.327.5973.1595 (2010).

21 Bonmassar, G., Fujimoto, K. & Golby, A. J. PTFOS: flexible and absorbable intracranial electrodes for magnetic resonance imaging. *PloS one* **7**, doi:10.1371/journal.pone.0041187 (2012).

22 Blythe, A. R. & Bloor, D. *Electrical properties of polymers*. 2nd edn, (Cambridge University Press, 2005).

23 Dang, Z. M., Wang, L., Yin, Y., Zhang, Q. & Lei, Q. Q. Giant Dielectric Permittivities in Functionalized Carbon-Nanotube/ Electroactive-Polymer Nanocomposites. *Adv Mater* **19**, 852-857, doi:10.1002/adma.200600703 (2007).

24 Acheson. Product Data Sheet Electrodag 423SS Graphite Based Polymer Thick Film Ink. (2005).

25 Acheson. Technical Datasheet Electrodag 479SS 2(2010).

26 Kato, H. *et al.* Composition of MRI phantom equivalent to human tissues. *Medical physics* **32**, 3199-3208 (2005).

27 Mattei, E. *et al.* Temperature and SAR measurement errors in the evaluation of metallic linear structures heating during MRI using fluoroptic probes. *Physics in medicine and biology* **52**, 1633-1646 (2007).

28 Medtronic. *Reference Manual - MRI guidelines for Medtronic Deep Brain Stimulation Systems.*, <http://professional.medtronic.com/pt/neuro/dbs-md/ind/mri-guidelines/#.VKwRYnsz_qQ> (2010 - Date of access: 27/01/2015).

29 Yeo, D. T., Wang, Z., Loew, W., Vogel, M. W. & Hancu, I. Local specific absorption rate in high-pass birdcage and transverse electromagnetic body coils for multiple human body models in clinical landmark positions at 3T. *Journal of magnetic resonance imaging : JMRI* **33**, 1209-1217, doi:10.1002/jmri.22544 (2011).

30 Medtronic. *Reference Manual - System Eligibility Battery Longevity - Neurostimulation systems for deep brain stimulation*, <http://manuals.medtronic.com/wcm/groups/mdtcom_sg/@emanuals/@era/@neuro/documents/documents/wcm_prod067815.pdf> (2010 - Date of access: 27/01/2015).

31 Toda, H. *et al.* A novel composite targeting method using high-field magnetic resonance imaging for subthalamic nucleus deep brain stimulation. *Journal of neurosurgery* **111**, 737-745, doi:10.3171/2008.12.JNS0861 (2009).

32 Ratner, B. D., Tyler, B. J. & Chilkoti, A. Analysis of biomedical polymer surfaces: polyurethanes and plasma-deposited thin films. *Clinical materials* **13**, 71-84 (1993).

33 Chu, C. C. Mechanical properties of suture materials: an important characterization. *Annals of surgery* **193**, 365-371 (1981).

34 Gonzalez, C. & Rodriguez, M. A flexible perforated microelectrode array probe for action potential recording in nerve and muscle tissues. *Journal of neuroscience methods* **72**, 189-195 (1997).

35 Hassler, C., von Metzen, R. P., Ruther, P. & Stieglitz, T. Characterization of parylene C as an encapsulation material for implanted neural prostheses. *Journal of biomedical materials research. Part B, Applied biomaterials* **93**, 266-274, doi:10.1002/jbm.b.31584 (2010).

36 Collins, C. M. & Smith, M. B. Signal-to-noise ratio and absorbed power as functions of main magnetic field strength, and definition of "90 degrees " RF pulse for the head in the birdcage coil. *Magnetic resonance in medicine : official journal of the Society of Magnetic Resonance in Medicine / Society of Magnetic Resonance in Medicine* **45**, 684-691 (2001).
